# Myocardial contractility and afterload in aortic stenosis

**DOI:** 10.1186/1532-429X-17-S1-P371

**Published:** 2015-02-03

**Authors:** Tazim Merchant, Nathaniel Reichek, Eddy Barasch, Madhavi Kadiyala, Meghana Jayam, Simcha Pollack, Jie J Cao, Alistair Young

**Affiliations:** 1 St. Francis Hospital,Research and Education Foundation, Roslyn, NY, USA; 2 Medicine, Stony Brook University, Stony Brook, NY, USA; 3 Biomedical Engineering, University of Pennsylvania, Philadelphia, NY, USA; 4 Applied Mathematics, St. John's University, New York City, NY, USA; 5 Anatomy, University of Auckland, Auckland, New Zealand; 6 Locust Valley High School, Locust Valley, NY, USA

## Background

In aortic stenosis(AS) with normal left ventricular ejection fraction(LV EF), circumferential and longitudinal systolic strains(CSt, LSt) are reduced but the underlying mechanisms are uncertain. Conventional indices of afterload(A) per unit of myocardium are suboptimal for strain analysis and based on inaccurate assumptions about myocardial properties, while conventional contractility(C) indices address LV chamber, not myocardial properties. We have shown that the product of end-systolic pressure(P) and volume(V), divided by LV mass(M), or (PV/M), is a more effective index of A than conventional wall stress(Mirsky, J. Biophys. 1969) for strain analysis, while the ratio of strain to PV/M is an effective strain-based index of C. These methods were used to evaluate the mechanism of reduced LV strain in AS with preserved EF.

## Methods

Age and gender matched patients with AS and normal volunteers(NL) were compared (Table). CMR was performed on a 1.5T scanner, using TrueFISP cine short and LV long axis imaging and volumetrics and feature-tracking CSt, LSt and strain rates evaluated(CIM, U. of Auckland). A was quantitated as LV end-systolic PV/M, and as circumferential and meridional wall stress(CWS,MWS). C was assessed using the ratios, CSt/(PV/M), LSt/(PV/M), CSt/CWS and LSt/MWS.

## Results

(Table 1): Mean EF was normal in both AS and NL but systolic CSt and LSt were reduced in AS, without significantly increased afterload. In contrast, contractility, expressed as CSt/(PV/M) and LSt/(PV/M) was reduced in AS, although wall stress based indices and strain rate(SR) were not. There were strong negative correlations between PV/M and EF in AS and NL and strong positive correlations between PV/M based contractile indices and EF, but regression slopes for strain/PV/M vs.EF and versus strain itself were significantly higher in AS, underscoring the role of reduced contractility. In a subset of AS patients(n=8), extracellular volume data were available but correction for smaller myocyte fraction of LV mass(72% AS vs 76% NL) did not significantly alter results.

**Table 1:** 

	NL	AS	P
AGE(yrs,(s.d.))	71.2(10.9)	71.8 (10.8)	ns

Gender	8M, 7F	8M, 7F	ns

EF(%)	58(6)	56(11)	ns

LVM(g)	89(23)	137(43)	<0.0001

LVP(mmHg)	138(12)	203(27)	<0.0001

Mean Gradient(mmHg)	X	49(18)	X

AVA(cm2)	X	0.8(0.2)	X

Global CStrain(%)	-19.7(2.7)	-16.5(4.4)	0.02

Global LStrain(%)	-15.6(2.4)	-12.2(3.2)	<0.003

PV/M(mmHgxml/g)	91.5(23.9)	98.6(34.8)	ns

CWStress (103dyn/cm2)	228.9(79.3)	202.3(81)	ns

MWStress (103dyn/cm2)	89.6(35.3)	63.9(39.4)	ns

CStrain/PV/M(%/mmHgxml/g)	-0.24(0.1)	-0.14(0.1)	0.05

LStrain/PV/M(%/mmHgxml/g)	-0.18(0.1)	-0.14(0.1)	0.03

CStrain/CWS(%/103dyn/cm2)	-0.1(0.04)	-0.1(0.06)	ns

LStrain/MWS(%/103dyn/cm2)	-0.2((0.01)	-0/3(0.06)	ns

R PV/M vs EF	-0.82	-0.82	<0.01 each

R CSt/PV/M vs EF	0.87	0.85	<0.00001 each

R LSt/PV/M vs EF	0.82	0.84	<0.00001 each

## Conclusions

Reduced systolic strain in AS with normal EF is due principally to reduced contractility, presumably due to known alterations in myocardial energetics, calcium handling and contractile proteins in hypertrophied myocardium. In contrast, differences in afterload are modest, so that overt "afterload excess" at the myocardial level is not seen, due to the adequacy of compensatory hypertrophy. In addition, the dependence of myocardial performance on contractile state is more marked in AS than in NL. Afterload and contractile indices based on PV/M were more useful than those based on conventional wall stress estimates.

## Funding

Supported by the St. Francis Research Foundation.

